# Middle lobe torsion after chest tube drainage for primary spontaneous pneumothorax: a case report

**DOI:** 10.1186/s44215-026-00268-z

**Published:** 2026-07-28

**Authors:** Hikaru Kohtake, Naoya Himuro, Katsutoshi Seto, Tetsuya Endo, Masashi Kobayashi

**Affiliations:** Department of Surgery, Division of Thoracic Surgery, Showa Medical University School of Medicine, 1-5-8 Hatanodai, Shinagawa-ku, Tokyo, 142-8666 Japan

**Keywords:** Pulmonary torsion, Lobar torsion, Pneumothorax, Chest tube drainage

## Abstract

**Background:**

Pulmonary torsion is an uncommon condition that occurs most frequently after pulmonary resection. Its occurrence in association with spontaneous pneumothorax is very limited. Herein, we report a rare case of pulmonary torsion that developed following chest tube drainage for primary spontaneous pneumothorax.

**Case presentation:**

A 26-year-old man with a history of chest drainage for right primary spontaneous pneumothorax presented to our emergency department with dyspnea. Chest radiography revealed a right tension pneumothorax, and chest drainage was immediately performed. Chest computed tomography (CT) revealed no apparent bullous disease, but atelectasis of the right middle lobe was observed. Despite the cessation of air leakage, surgical intervention was planned on hospital day 3 because of the recurrence of spontaneous pneumothorax and its presentation as tension pneumothorax. Intraoperatively, the right middle lobe was rotated to the dorsal side of the lower lobe. No congestion or ischemic changes were observed, and detorsion was successfully performed. No obvious bullous disease was detected, though pleural changes at the lung apex were ablated and covered with a polyglycolic acid sheet. The postoperative course was uneventful, and the patient was discharged on postoperative day 4.

**Conclusions:**

Chest tube drainage for primary spontaneous pneumothorax is a routine procedure widely performed in emergency medicine. Therefore, clinicians must recognize that pulmonary torsion, a potentially fatal complication, can occur unexpectedly in the context of this common procedure. When lobar atelectasis is observed after chest tube insertion, clinicians should consider pulmonary torsion and promptly perform CT imaging.

## Background

Pulmonary torsion is a rare but life-threatening condition, defined as the rotation of the lung parenchyma on its bronchovascular pedicle, resulting in vascular compromise and airway obstruction. The overall incidence is extremely low, ranging from 0.089% to 0.4% [1]. It most commonly occurs after pulmonary resection, whereas its association with spontaneous pneumothorax is rare, with only two reported cases to date [2, 3]. Herein, we report a rare case of right middle lobe torsion following chest tube drainage for primary spontaneous pneumothorax.

## Case presentation

**Fig. 1:**
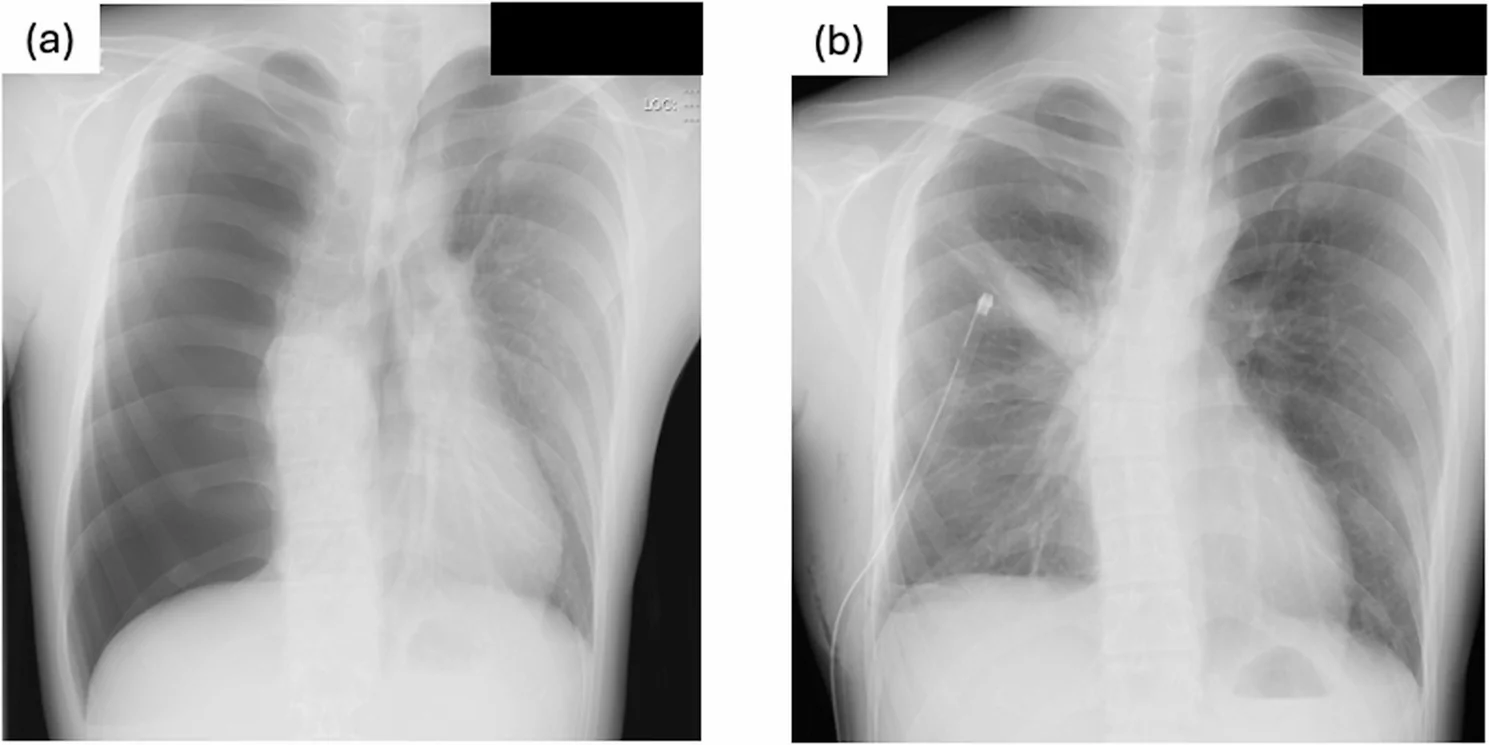
**a** Chest radiograph showing complete collapse of the right lung with mediastinal shift to the left. **b** On the day after chest tube drainage, lung re-expansion was obtained, but cord-like atelectasis was observed in the right middle lung field

A 26-year-old man who had undergone chest tube drainage for right spontaneous pneumothorax 7 months prior presented to our emergency department with a 1-day history of acute dyspnea and chest pain. Chest radiography revealed complete collapse of the right lung with a contralateral mediastinal shift, leading to a diagnosis of tension pneumothorax (Fig. 1a). Immediate tube thoracostomy was performed by emergency physicians, after which the patient was referred to our department and admitted. At presentation, vital signs were as follows: heart rate, 113 beats/min; blood pressure, 121/88 mmHg; respiratory rate, 20 breaths/min; SpO₂, 95% in room air; and body temperature, 36.8 °C. Laboratory findings demonstrated leukocytosis (white blood cell count, 13,400/µL) with a C-reactive protein of 0.04 mg/dL.

**Fig. 2:**
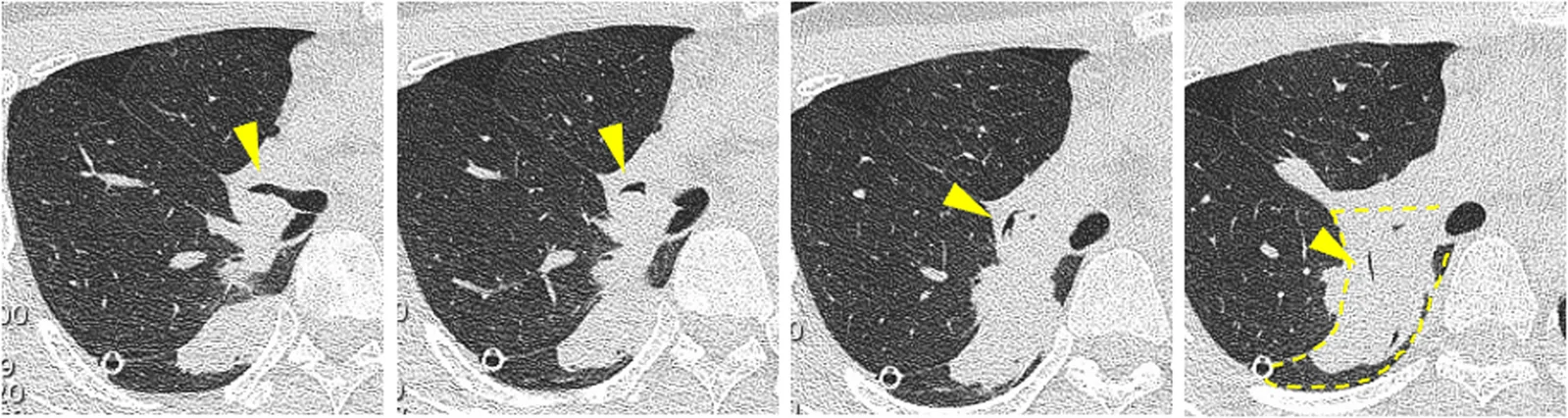
Chest computed tomography scan showed no obvious bullae. Atelectasis of the right middle lobe was observed, and the middle lobe bronchus extended posteriorly (arrow), indicating torsion of the middle lobe. The outline of the middle lobe is indicated by the dotted line

**Fig. 3:**
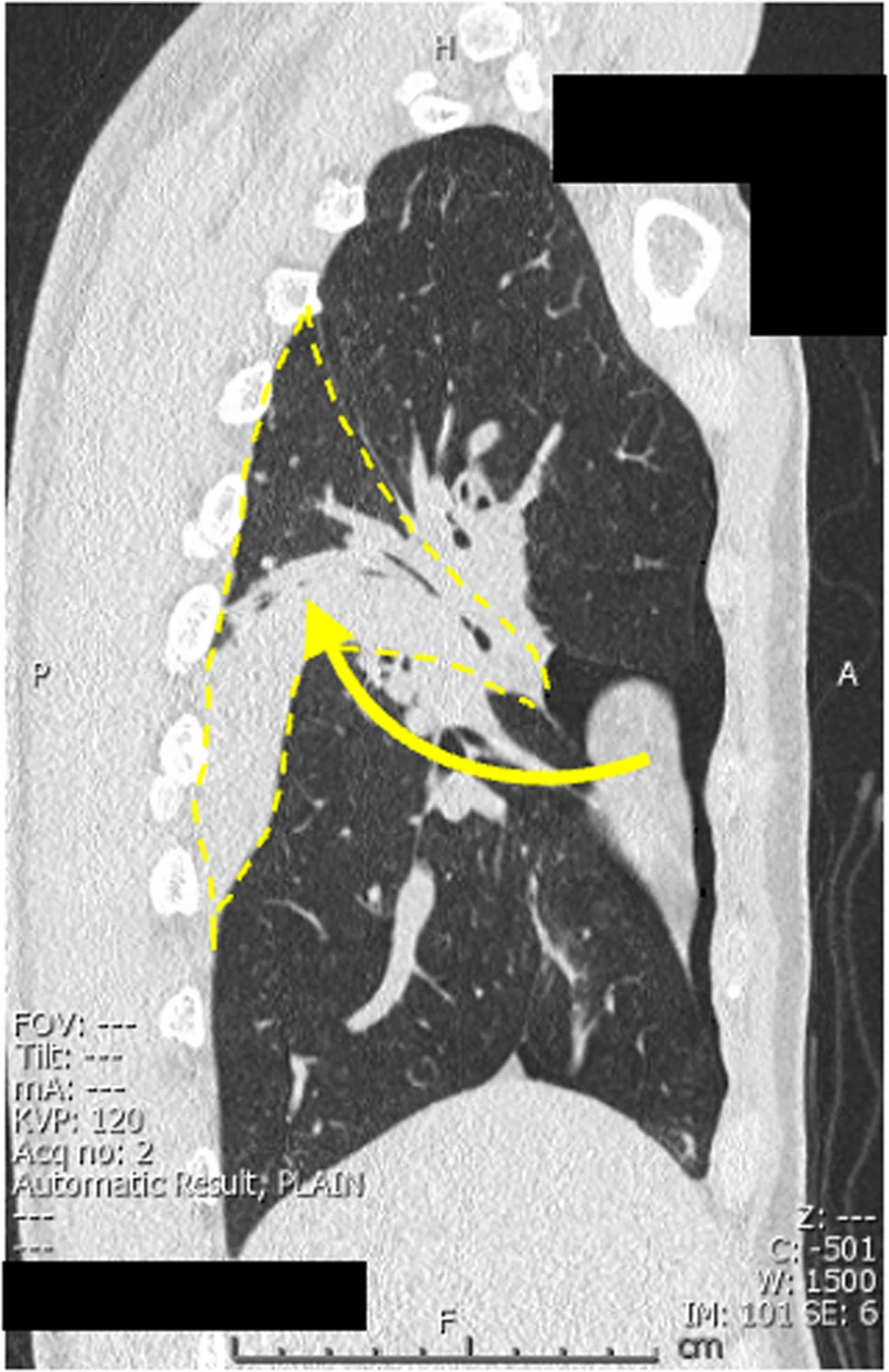
Sagittal chest computed tomography scan showed the right middle lobe with atelectasis, positioned in a dorsal direction (arrow). The outline of the middle lobe is indicated by the dotted line. The right side of the image corresponds to the ventral direction

Chest radiography performed on the day following admission confirmed adequate lung re-expansion (Fig. 1b) with no air leaks. Chest CT performed to evaluate for pulmonary cysts demonstrated no obvious bullae or blebs; however, right middle lobe atelectasis was observed, with the right middle lobe bronchus coursing posterior to the hilum (Figs. 2 and 3). The indwelling chest tube was positioned dorsal to the torsed middle lobe. At the time, this bronchial deviation was not recognized, and the atelectasis was attributed to the preceding pneumothorax. The patient presented with tension pneumothorax, and owing to the risk of spontaneous pneumothorax recurrence, surgical intervention was planned. As pulmonary torsion had not been suspected, the surgery was scheduled on an elective basis on hospital day 3.

**Fig. 4:**
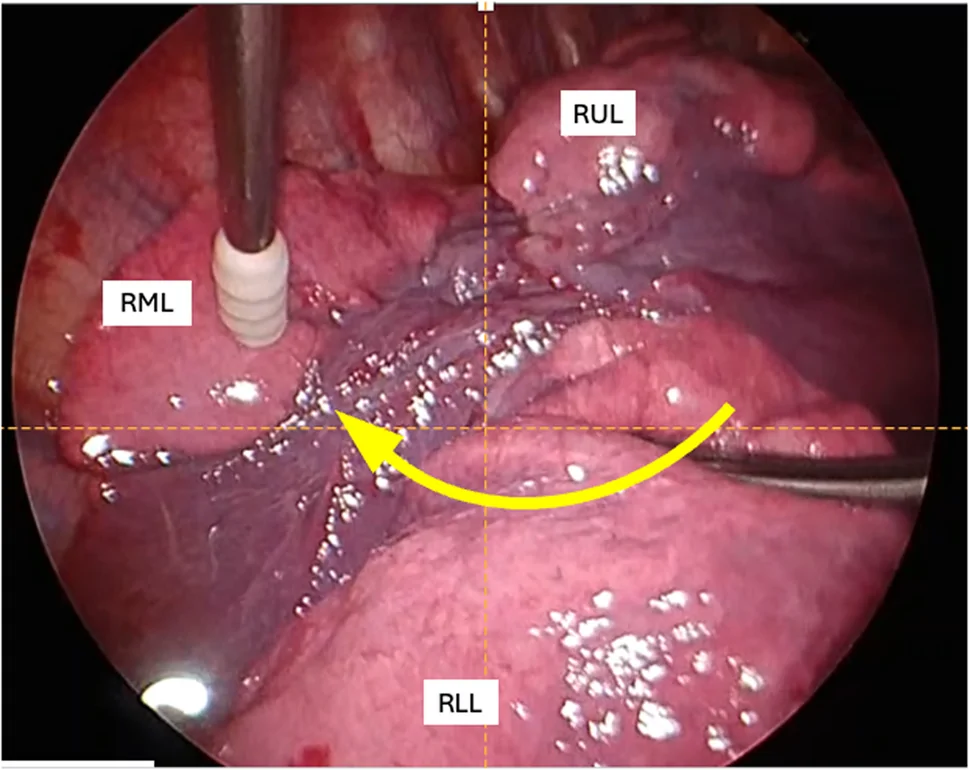
Intraoperative view showing a completely developed interlobar fissure. The collapsed right middle lobe was displaced and located posterior to the lower lobe and rotated approximately 90 degrees clockwise (arrow). No congestion or ischemic changes were observed. In this view, the cranial side is at the top and the ventral side is to the right

The surgery was performed under general anesthesia with one-lung ventilation in the left lateral decubitus position. Thoracoscopic examination revealed a complete pulmonary fissure. The collapsed right middle lobe was displaced dorsally to the lower lobe and rotated approximately 90 degrees clockwise, consistent with lobar torsion (Fig. 4). The parenchyma retained a normal pink color without venous congestion or ischemic changes. Thus, the lobe was considered viable, and detorsion alone was performed without resection. A leak test demonstrated no air leakage, and systematic inspection of the entire lung surface revealed no identifiable pulmonary bullae or blebs. Pleural changes extending from the mediastinal surface to the lung apex were observed and were ablated using soft coagulation. Polyglycolic acid sheets were applied to the ablated areas and placed over the surface of the upper and middle lobes to prevent retorsion.

The postoperative course was uneventful. Radiography confirmed the resolution of atelectasis and adequate lung re-expansion. The chest tube was removed on postoperative day 3, and the patient was discharged on day 4 without complications.

## Discussion and conclusions

Pulmonary torsion is an uncommon condition, and most reported cases occur postoperatively. It is typically described in three clinical circumstances: postoperative, spontaneous, and posttraumatic. According to a systematic review [4], the majority of cases occur postoperatively (72.8%) followed by spontaneous (21.6%) and posttraumatic (5.6%) incidences. Among spontaneous cases, pulmonary torsion has been associated with underlying conditions such as massive pleural effusion, lobar atelectasis, and pneumothorax. A limited number of pulmonary torsion cases associated with spontaneous pneumothorax have been reported, with only two previously reported cases (Table 1) [2, 3]. This case presents an extremely rare clinical course in which pulmonary torsion occurred following chest drain insertion for primary spontaneous pneumothorax.

**Table 1:** Clinical characteristics of the three reported cases of pulmonary torsion associated with spontaneous pneumothorax, including two previously published cases and the present case

Author	Age /Sex	Pneumothorax type	Affected lobe	Symptoms	History of chest tube drainage	Diagnosis	Management
Berkmen	63/F	SP	RUL	Chest pain /dyspnea	-	X-ray	Chest tube drainage
Stefanidis	69/M	SSP (COPD)	Whole lung	Dyspnea	+	CT	Surgical detorsion
Our case	26/M	PSP	RML	Asymptomatic	+	CT	Surgical detorsion

*SP* spontaneous pneumothorax, *SSP* secondary spontaneous pneumothorax, *PSP* primary spontaneous pneumothorax, *COPD* chronic obstructive pulmonary disease, *CT* computed tomography, *RML* right middle lobe, *RUL* right upper lobe

Several factors contribute to the pathogenesis of pulmonary torsion. Felson et al. identified five risk factors: (1) atelectasis, (2) a long lobar pedicle, (3) a complete interlobar fissure, (4) pneumothorax or pleural effusion, and (5) division of the pulmonary ligament [5]. In the present case, complete fissures were observed intraoperatively and the middle lobe was found to be rotated behind the lower lobe. Two possible mechanisms are proposed to account for the torsion in this case: first, direct physical displacement of the middle lobe caused by contact with the chest tube during its insertion, and second, mechanical obstruction of normal middle lobe re-expansion by the indwelling chest tube, preventing the lobe from returning to its anatomical position. The presence of a complete fissure, as confirmed intraoperatively, likely conferred increased mobility to the middle lobe and may have further predisposed it to torsion.

Diagnosing pulmonary torsion is often difficult, and its typical symptoms and physical signs are frequently absent. According to previous reports, symptoms associated with pulmonary torsion include chest pain, fever, and cough; however, 20% of patients are asymptomatic [1]. In our case, lung expansion after chest tube drainage was adequate, blood tests showed no elevation in C-reactive protein levels, and the patient had no notable subjective symptoms. Chest radiography revealed only linear atelectasis in the right middle lung field. However, chest CT showed that the right middle lobe bronchus extended posterior to the hilum, suggesting middle lobe torsion.

The treatment of pulmonary torsion consists of either resection of the torsed lobe or surgical detorsion. If a torsed pulmonary lobe leads to necrosis, lobectomy or ligation of the pulmonary veins draining the torsed lobe before pulmonary detorsion is recommended. The rationale behind these treatments is that inflammatory mediators and thromboembolic substances released from necrotic tissue can spread throughout the body, potentially causing serious complications such as acute respiratory distress syndrome, multiple organ failure, and cerebral infarction [1, 4]. In the present case, the color of the lung parenchyma was preserved, and no congestion was observed; therefore, detorsion alone was performed.

To prevent recurrent torsion after detorsion, fixation of the affected lobe to an adjacent lobe by suturing has been reported, mainly for postoperative pulmonary torsion [1]. In the present case, however, the causative factors—the pneumothorax and the indwelling chest tube—had been eliminated, and the detorsed middle lobe remained viable and re-expanded well. Therefore, suture fixation was considered unnecessary. Coverage with polyglycolic acid sheets is not an established method of torsion prophylaxis and may be insufficient when used alone; nevertheless, the sheets were applied over the upper and middle lobes as an adjunct to limit excessive mobility of the middle lobe.

The present case is a rare instance of right middle lobe torsion occurring following chest tube drainage for primary spontaneous pneumothorax. Chest tube drainage for pneumothorax is a commonly encountered procedure in clinical practice; however, even when satisfactory lung re-expansion is achieved and air leak has ceased, the possibility of pulmonary torsion should be considered if atelectasis is identified on chest radiography. In such cases, a prompt chest CT should be performed to exclude this potentially fatal complication.

## Data Availability

Not applicable.

## Abbreviation

CT: Computed tomography
